# The relationship between polycystic ovary syndrome, glucose tolerance status and serum preptin level

**DOI:** 10.1186/1477-7827-10-10

**Published:** 2012-02-06

**Authors:** Zhiqin Bu, Kakei Kuok, Jie Meng, Rui Wang, Bei Xu, Hanwang Zhang

**Affiliations:** 1 Reproductive Medicine Center, Tongji Hospital, Tongji Medical College, Huazhong University of Science and Technology, Wuhan, People's Republic of China

## Abstract

**Background:**

Polycystic ovary syndrome (PCOS) is linked to obesity, impaired glucose tolerance and diabetes. Recently, studies have found that preptin enhances insulin secretion in rats and might play a role in the pathogenesis of diabetes and PCOS in humans. The aim of this study was to evaluate the relationship between PCOS, glucose tolerance status, and serum preptin level.

**Methods:**

This study was conducted in a university-affiliated hospital from October 2010 to August 2011. Anthropometric parameters, sex hormone concentrations, blood pressure, lipid profiles, fasting glucose and insulin, 2-h blood glucose after glucose overloaded (2hOGTT), glycosylated haemoglobin (HbA_1_c), homeostasis model assessment-insulin resistance index (HOMA-IR), and serum preptin of the samples were analyzed.

**Results:**

Sixty-three PCOS patients, including 33 women with normal glucose tolerance (NGT) and 30 women with impaired glucose tolerance (IGT), and 63 patients without PCOS, including 35 women with NGT and 28 women with IGT were recruited in this study. For patients with and without PCOS, women with IGT had higher serum preptin levels compared with women with NGT. Preptin levels in PCOS patients were higher compared with patients without PCOS, but the difference was not significant. Fasting serum preptin levels correlated positively with TG, SBP, DBP, FBG, 2hOGTT, and HOMA-IR in simple regression analysis of the pooled data. While in multiple stepwise regression analysis, preptin levels were independently related with glucose tolerance, but not with PCOS.

**Conclusions:**

Irrespective of PCOS status, women with IGT had higher serum preptin levels compared with women with NGT. Preptin levels are related with glucose tolerance status, but not with PCOS status.

## Background

Polycystic ovary syndrome (PCOS) is an endocrine disorder associating with female infertility due to anovulation [1]. PCOS is characterized by chronic anovulation and hyperandrogenism, either in the form of biochemical androgen excess or clinically as hirsutism, acne, and/or male pattern alopecia. Moreover, PCOS has been linked to obesity, type 2 diabetes mellitus (T2DM), dyslipidemia, hypertension, and heart disease [2-4]. Although PCOS was described more than 50 years ago, the underlying cause of the disorder is still unclear. Recently, many studies have documented the presence of insulin resistance (IR) in both obese and lean PCOS patients, and some investigators consider IR to be an important risk factor for the development of the metabolic syndrome in women with PCOS [5].

Preptin is a recently isolated 34-amino acid peptide hormone corresponding to Asp^69^-Leu^102 ^of the proinsulin-like growth factor II E-peptide. Preptin is present in islet β-cell granules and is co-secreted with insulin in response to glucose. Recent studies have shown that preptin enhances insulin secretion in rats [6], and there is a potential association between preptin and insulin resistance in humans [7].

As is well-known, many PCOS women have IR, impaired glucose tolerance (IGT), and are even diagnosed with T2DM. Women with PCOS have been considered to be insulin resistant, and are at markedly high risk for developing diabetes. According to some clinical studies, the prevalence of IGT and T2DM in PCOS women is 31%-35% and 7.5%-10.0%, respectively [8,9]. Thus, the objectives of the current study were to determine serum preptin levels in women with PCOS, and to examine the relationship between preptin levels and PCOS and glucose tolerance with and without adjusting for confounders.

## Methods

### Subjects and study design

Sixty-three patients with PCOS (33 patients with NGT and 30 patients with IGT) and 63 patients without PCOS (35 patients with NGT and 28 patients with IGT) participated in the study. The PCOS patients were recruited from our infertility clinic, and the patients without PCOS were recruited during routine medical check-ups. Institutional Review Board (IRB) approved the study, and informed consent was obtained from all participants.

The diagnosis of PCOS was confirmed using the Rotterdam criteria, including at least two of the following: oligo- or anovulation, clinical or biochemical signs of hyperandrogenism, and polycystic ovaries on ultrasound; other endocrinopathies were excluded [10]. The diagnosis of IGT was based on oral glucose tolerance tests and World Health Organization criteria [11]. All the patients had not been treated with insulin sensitizers, ovulation induction medication, oral antihyperglycaemic agents, insulin or other drugs.

Body mass index (BMI) was calculated as the ratio of weight (Kg) to height squared (m^2^). Waist and hip circumferences (cm) were measured in duplicate with an anthropometric tape while the subjects were wearing light clothing. Waist circumference was measured at the minimum circumference between the iliac crest and the rib cage. Hip circumference was measured at the maximum protuberance of the buttocks, and the WHR was calculated. Blood pressure was measured on the right arm, with the patients sitting in a chair.

The blood of patients was routinely obtained in the morning following an overnight fast on day 3 of a natural menstrual cycle. Plasma was collected and frozen at -80°C until assayed. The concentration of fasting serum preptin was determined by ELISA (Phoenix Pharmaceuticals Inc., Belmont, CA, USA). Serum follicle-stimulating hormone (FSH), luteinizing hormone (LH), total testerone (T), and insulin were measured by radioimmunoassay (RIA) (Biotechnology Institute of the North, Beijing, China). The inter-assay and intra-assay coefficients of variation were 6.3% and 10.8% for FSH, 7.1% and 11.4% for LH, 5.8% and 11.0% for insulin. Fasting glucose was determined using the glucose oxidase method. HbA_1_c was measured by isoelectric focusing. Serum TG, TC, HDL-C and LDL-C levels were measured by routine methods. IR was estimated via the homeostasis model assessment insulin resistance index (HOMA-IR), as follows: HOMA-IR = fasting insulin (mU/L) × fasting glucose (mmol/L)/22.5.

### Statistics analysis

SPSS software (version 13.0, SPSS Inc, Chicago, IL) was used for statistical analysis. Comparisons between groups were performed using the Mann-Whitney U test because the data were not normally distributed. The Spearman order correlation coefficients were used to determine the relationships between the preptin level and biochemical and demographic values. A logistic regression model was performed to determine the predictive effect of serum preptin level on IGT and PCOS. A *P *value < 0.05 was considered statistically significant.

## Results

**Figure 1:**
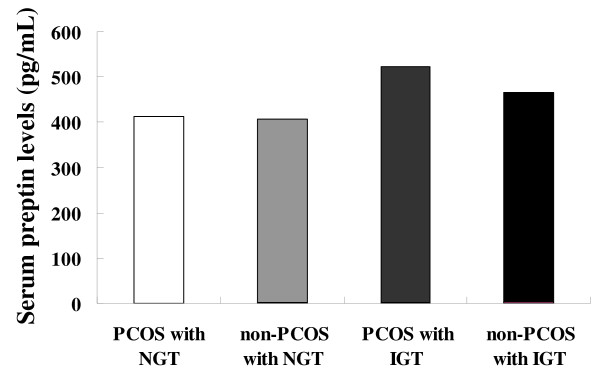
**Serum preptin levels by polycystic ovary syndrome (PCOS) and glucose tolerance status**. For patients with and without PCOS, women with IGT had higher preptin levels than women with NGT. However, preptin levels were comparable in 63 patients with PCOS compared with 63 patients without PCOS. IGT, impaired glucose tolerance; NGT, normal glucose tolerance; PCOS, polycystic ovary syndrome.

Patient characteristics according to PCOS and glucose tolerance status were shown in Table 1. PCOS patients with IGT had higher TG (*P *< 0.01), TC (*P *< 0.05), and LDL-C levels (*P *< 0.01), and SBP (*P *< 0.01) and DBP (*P *< 0.05) than PCOS patients with NGT. Compared with PCOS patients with NGT, PCOS patients with IGT also had higher fasting glucose (*P *< 0.01), 2h_OGTT glucose (*P *< 0.01), HbA_1_c (*P *< 0.01), and HOMA-IR (*P *< 0.01) levels. Patients without PCOS and IGT had higher fasting insulin levels (*P *< 0.01) than patients without PCOS and NGT. We also noted significantly higher fasting glucose (*P *< 0.01), 2h_OGTT glucose (*P *< 0.01), HbA_1_c (*P *< 0.01), and HOMA-IR (*P *< 0.01) levels in IGT patients without PCOS compared with NGT patients without PCOS. The serum preptin levels were higher in patients with IGT compared with NGT controls in the sub-groups with and without PCOS. However, no significant difference in preptin levels existed when the 63 PCOS patients were compared with the 63 patients without PCOS. The preptin level was highly dependent on the glucose tolerance status, but not on PCOS (Figure 1). In addition, basing on simple regression analysis of the pooled data, we found that the fasting preptin level was positively correlated with the TG, FBG, 2h_OGTT, and HOMA-IR levels, and SBP and DBP.

**Table 1:** Clinical characteristics and laboratory values of study sample according to polycystic ovary syndrome (PCOS) and glucose tolerance status

	PCOS		Non-PCOS	
	**NGT**	**IGT**	**NGT**	**IGT**

**No**.	33	30	35	28

**Age (y)**	26.1 ± 2.4	27.2 ± 3.1	28.4 **± **3.1	29.4 ± 3.8

**BMI (kg/m^2^)**	21.8 ± 2.7	24.5 ± 3.1^b^	21.7 ± 3.2	20.6 ± 2.6

**WHR**	0.9 ± 0.6	0.9 ± 0.6	0.9 ± 0.7	0.9 ± 0.9

**FSH (mIU/mL)**	6.3 ± 1.6	5.5 ± 1.5^a^	6.3 ± 1.6	7.0 ± 3.9

**LH (mIU/mL)**	9.9 ± 3.9	8.6 ± 7.1	6.4 ± 2.0	4.3 ± 2.0^d^

**Total T (ng/dL)**	46.4 ± 11.7	47.5 ± 17.0	39.8 ± 10.0	36.0 ± 13.1

**TG (mmol/L)**	1.2 ± 0.5	1.9 ± 0.6^b^	1.3 ± 0.5	1.2 ± 0.6

**TC (mmol/L)**	4.4 ± 0.6	4.8 ± 0.6^a^	4.2 ± 0.8	4.2 ± 0.8

**HDL-C (mmol/L)**	1.6 ± 0.3	1.2 ± 0.2^b^	1.50 ± 0.3	1.4 ± 0.5

**LDL-C (mmol/L)**	2.4 ± 0.5	2.7 ± 0.2^b^	2.6 ± 0.4	2.3 ± 0.5^c^

**SBP (mmHg)**	113.8 ± 14.9	125.9 ± 13.6^b^	123.7 ± 13.3	112.2 ± 15.1^d^

**DBP (mmHg)**	77.1 ± 9.8	82.9 ± 11.4^a^	76.9 ± 8.0	78.0 ± 10.7

**FBG (mmol/L)**	5.5 ± 0.5	6.5 ± 0.8^b^	5.3 ± 0.4	5.8 ± 0.7^d^

**2h_OGTT (mmol/L)**	7.0 ± 0.5	9.2 ± 0.8^b^	7.1 ± 0.5	9.8 ± 1.0^d^

**FIns (mU/L)**	12.0 ± 3.7	13.6 ± 4.8	10.3 ± 1.5	13.6 ± 5.3^d^

**HbA_1_c (%)**	5.3 ± 0.4	6.0 ± 0.9^b^	5.5 ± 0.2	6.0 ± 0.7^d^

**HOMA-IR**	2.9 ± 0.9	3.9 ± 1.5^b^	2.4 ± 0.4	3.5 ± 1.4^d^

**Preptin (pg/mL)**	413.7 ± 106.6	522.1 ± 135.1^b^	406.1 ± 79.9	466.4 ± 121.1^c^

Data presented as mean ± SD.*PCOS *polycystic ovary syndrome, *NGT *normal glucose tolerance, *IGT *impaired glucose tolerance, *BMI *body mass index, *WHR *waist-to-hip ratio, *FSH *follicle-stimulating hormone, *LH *luteinizing hormone, *total T *total testerone, *TG *triglyceride, *TC *total cholesterol, *HDL *high-density lipoprotein, *LDL *low-density lipoprotein; *SBP *systolic blood pressure, *DBP *diastolic blood pressure, *FBG *fasting blood glucose; *2h_OGTT *2-h blood glucose after glucose overload, *FIns *fasting insulin, *HbA1c *glycosylated haemoglobin, *HOMA-IR *homeostasis model assessment-insulin resistance index, *HOMA-IS *homeostasis model assessment-β-cell insulin secretion index

a *P *< 0.05 PCOS patients with IGT compared with PCOS patients with NGT

b *P *< 0.01 PCOS patients with IGT compared with PCOS patients with NGT

c *P *< 0.05 Non-PCOS patients with IGT compared with non-PCOS patients with NGT

d *P *< 0.01 Non-PCOS patients with IGT compared with non-PCOS patients with NGT

As shown in Table 2, we evaluated the separate and joint effects of preptin and other variables on PCOS and IGT. In model 1, while IGT and PCOS were dependent variables, preptin was included as a continuous independent variable alone; preptin had a significant effect on IGT, but not on PCOS. From model 2 to model 5, when IGT and PCOS were also dependent variables, after controlling for anthropometric variables, age, blood pressure, sex hormone concentrations, and lipid profiles, there was a significant effect of preptin on IGT, but not on PCOS.

**Table 2:** Association of serum preptin levels with IGT and PCOS in adjusted models

	IGT			PCOS		
	**OR**	**95% CI**	***P *value**	**OR**	**95% CI**	***P *value**

***Model 1***						

**Preptin**	1.008	1.004-1.012	< 0.001	1.002	0.998-1.006	0.134

***Model 2***						

**Preptin**	1.008	1.004-1.012	< 0.001	1.002	0.999-1.006	0.156

**Age**	--	--	--	--	--	--

***Model 3***						

**Preptin**	1.008	1.004-1.013	< 0.001	1.002	0.998-1.006	0.346

**Age, BMI, WHR, BP**	--	--	--	--	--	--

***Model 4***						

**Preptin**	1.011	1.005-1.016	< 0.001	1.002	0.997-1.007	0.477

**Age, BMI, WHR, BP, sex hormone**	--	--	--	--	--	--

***Model 5***						

**Preptin**	1.012	1.006-1.018	< 0.001	1.002	0.997-1.007	0.430

**Age, BMI, WHR, BP, sex hormone, lipid profiles**	--	--	--	--	--	--

*BMI *body mass index, *WHR *waist-to-hip ratio, *BP *blood pressure, including systolic blood pressure and diastolic blood pressure; sex hormone = including FSH, LH and total T; lipid profiles = including TC, TG, HDL-C and LDL-C

## Discussion

Preptin was first purified and isolated from cultured murine βTC6-F7 β-cells by Buchanan et al. in 2001. Buchanan et al. reported that the second phase of glucose-mediated insulin secretion was increased by 30% in isolated, perfused rat pancreas which were infused with preptin. In addition, the binding of endogenous preptin by anti-preptin antibodies decreased insulin secretion. These findings supported the view that preptin enhances insulin secretion in rats *in vitro *[6].

Subsequently, preptin levels were shown to be higher in patients with D2TM and IGT compared with healthy controls. Fasting plasma preptin was positively correlated with HOMA-IR, but not with insulin, suggesting that preptin may play a role in the pathogenesis of IR without affecting insulin secretion [7]. Interestingly, another study assessed maternal serum and cord blood preptin levels in gravidas with gestational diabetes mellitus [12]. Both studies showed that preptin may have an association with diabetes mellitus.

To date, few published trials have been conducted focusing on the significance of preptin in humans, and only study was conducted in PCOS patients. Celik and colleagues reported that serum preptin levels in patients with PCOS were higher compared with patients without PCOS, suggesting that preptin is involved in the pathogenesis of PCOS [13].

The findings of Celik et al. were new and very interesting. Yet our main concern was the true reason for high levels of serum preptin in patients with PCOS. We are aware that many patients with PCOS have IGT or D2TM, both of which can cause an elevation of preptin in serum. Thus, we raised a hypothesis that PCOS was not associated with elevation of serum preptin.

To determine the preptin levels in PCOS patients and the net relationship between PCOS, glucose tolerance status, and the preptin level, we designed the current study as follows. First, we divided the samples into PCOS and non-PCOS groups. Then, we measured the preptin levels in women with IGT and NGT in each group. PCOS patients with IGT had significantly higher preptin levels compared with PCOS patients with NGT. Similarly, serum preptin levels were higher in patients without PCOS and IGT. However, when the preptin levels in all 126 patients were compared, no significant difference existed between patients with and without PCOS, which were not consistent with the results of Celik et al., thus indicating that the relationship between PCOS and preptin is very poor. Furthermore, we investigated the preptin levels in women with and without PCOS and NGT, and there were no differences between these two groups, which made our hypothesis more credible. We also used multivariate logistic regression analysis to explore the association between serum preptin levels and PCOS and glucose tolerance status, and observed that IGT was highly dependent on preptin, while PCOS had a weak association with preptin. Moreover, adjustment for anthropometric variables (blood pressure, and sex hormone and lipid profiles) did not affect the relationship between preptin and PCOS and glucose tolerance status. All of these findings led us to conclude that it is IGT, but not PCOS, that is responsible for the elevation of serum preptin levels in women with and without PCSO.

Another interesting finding of the current study based on simple regression analysis of pooled data was that serum preptin levels are positively correlated with HOMA-IR, but not with fasting insulin, indicating that there is a potential link between IR and preptin. Currently we are attempting to determine the relationship between preptin and IR in women with PCOS by noting the preptin changes before and after administration of insulin sensitizers.

Because this was a preliminary study focusing on PCOS, glucose tolerance status and preptin, and there were no T2DM patients in the current study, more studies including both IGT and T2DM patients are needed to clarify our findings.

## Conclusions

The results of our study showed that preptin levels were elevated in IGT patients compared with NGT patients, and the serum preptin levels were comparable in patients with and without PCOS. Serum preptin levels are related with glucose tolerance status, but not with PCOS status.

## Competing interests

The authors declare that they have no competing interests.

## Authors' contributions

ZB, KK and JM were responsible for the study design and manuscript writing. RW collected and analyzed the data. BX and HZ supervised this whole study. All authors read and approved the final manuscript.
